# Sparking creativity: Encouraging creative idea generation through automatically generated word recommendations

**DOI:** 10.3758/s13428-024-02463-8

**Published:** 2024-07-16

**Authors:** Talia A. Wise, Yoed N. Kenett

**Affiliations:** 1 Faculty of Data and Decision Sciences, Technion – Israel Institute of Technology, 3200003 Haifa, Israel; 2 Department of Information Science, Cornell University, Ithaca, NY USA

**Keywords:** ·Creativity, Semantic networks, Word recommendations, Expertise

## Abstract

Creative block is a familiar foe to any who attempt to create and is especially related to “writers block”. While significant effort has been focused on developing methods to break such blocks, it remains an active challenge. Here, we focus on the role of semantic memory structure in driving creative block, by having people get “stuck” in a certain part of their semantic memory network. We directly examine whether we can “pull out” a participant from where they got “stuck” in their semantic memory, breaking their creative impasse. Our Associative Creativity Sparker (ACS) is a cognitive network science-based online tool that aims to spark creative ideas and break creative impasse: Once a participant runs out of ideas in a creative idea generation task, word recommendations are suggested to prime new ideas. These word recommendations are either *towards* or *away* from previous ideas, as well as *close* or *far* from the target object, based on a conceptual space extracted from the participants responses using online text analysis. In Study 1, 121 participants use the ACS to generate creative alternative uses for five different objects and completed creativity and *Gf* tasks. In Study 2, we repeat the design of Study 1, but further examine the impact of writing experience on the ACS, by examining 120 novice and 120 experienced writers. Across both studies, our results indicate that the location of word recommendations affects the fluency and originality of one’s ideas, and that novice and experienced writers differently benefit from these word recommendations.

## Introduction

The concept of “writer’s block” was introduced by psychoanalyst Edmund Bergler (Bergler, 1950), but one must only look to the classic invocation to the muses in Greek and Latin poetry to know that writer’s block has been part of the writing process for much longer. Creative block is a common part of people's creative process, and is a source of negative emotion, distress and even loss of income (Ahmed & Güss, 2022). Creative block is nonetheless associated with creative achievement and might be caused by a positive desire for renewal – at the breakthrough to the creative impasse lies the ‘aha’ moment of inspiration and discovery (Crosson, 1982). Methods that unblock creativity may contribute positively to wellbeing and productivity, and to our understanding of the creative process. Developing cognitive theories of how creativity tools and methods may best support the human creative process is especially important now that an abundance of AI-based creativity and writing assistance tools are becoming widely available. The current research presents the Associative Creativity Sparker (ACS) method for unblocking creativity, based on recent advances in computational methods and research on the role of semantic memory in the creative process (Kenett, 2024)﻿. 

Previous work on breaking a creative impasse has examined group brainstorming (Nijstad & Stroebe, 2006), crowdsourced suggestions (Chan et al., 2017; Siangliulue et al., 2015), sleeping and incubation periods (Sio et al., 2013; Sio & Rudowicz, 2007), subliminal messaging (Gonçalves et al., 2017), and hypnotically inducing dreams (Davé, 1979). Such methods – that aim to break creative impasse – are often based on techniques of successful writers, such as seeking inspiration through reading or discussion, which may be resource-demanding and are harder to study from a cognitive perspective. Current cognitive methods for breaking through creative blocks are aimed at the entire creative process, and regard impasse-break as a side-effect of improving creativity (Ahmed & Güss, 2022).

Given that creative block is related to “being stuck” and the effort to “get out” of such a block, semantic memory may play a critical role in such phenomena. Semantic memory is the cognitive system that stores facts and knowledge (Kumar, 2021) and plays a central role in creative ideation (Abraham & Bubic, 2015; Benedek et al., 2023; Kenett, 2024). The associative theory of creativity posits that creativity is related to the ability to form associations between distant or unrelated concepts in semantic memory (Beaty & Kenett, 2023; Mednick, 1962). This suggests that offering distant concepts to someone experiencing creative block might allow them to come up with new creative ideas. Following this theory, various creativity support methods incorporate idea suggestions to encourage creative associations (Chan et al., 2017; Siangliulue et al., 2015). Our study moves a step forward in this direction, by combining recent advances in computational modeling to represent semantic memory structure using online text analysis methods coupled with graph theory methodologies. This allows us to study why certain ideas might re-inspire creativity after impasse, while other ideas might help less. Our method specifically addresses the cognitive component of creative block – which includes fixation on a specific idea or rigid thinking (Ahmed & Güss, 2022; Chrysikou et al., 2016; Duncker & Lees, 1945; Schultz & Searleman, 2002).

Existing research on writer’s block focuses and sometimes even questions the existence of a “blocked writer,” who has prolonged and extremely severe instances of complete inability to write (Kaufman & Kaufman, 2009). This type of block has been associated more strongly with mental health issues such as depression, anxiety, or general life difficulties (e.g., Herz et al., 2020). In general, creative block is considered on a short-term and smaller-scale level of supporting creativity of individual ideas, or of small sections of text. Especially, this form of writer’s block has been found to occur in the vast majority of writers (Ahmed & Güss, 2022). In these cases, block due to cognitive causes such as perfectionism and rigid thinking is more associated with the idea generation phase of writing compared to writer’s block due to motivational causes which was found to occur more in the articulation phase of writing. Eminent authors have reported distinct writing phases aimed at facilitating idea generation and its translation to actual texts (Kaufman & Kaufman, 2009): a free-writing phase where everything that comes to mind is written down to promote improvisational creativity, and an analytical writing phase to organize, articulate and develop the aesthetic qualities of the text (Lubart, 2009).

There has been a recent influx of research in text-based creativity support systems (Calderwood et al., 2020; Huang et al., 2020; Lee et al., 2022). Many works examine the effects of conceptual distance of suggestions on the quality of generated ideas (Chan et al., 2017; Rhys Cox et al., 2021). In an idea generation task, semantically far inspirations were found to be harmful during the productive ideation phase, potentially even causing creative fixations, while they were not harmful during impasse (Chan et al., 2017). In a study on the effects of common examples on creative fixation in an alternative uses task, it was found that showing common uses to participants reduced their creativity, but when instructions to avoid the common uses were added, participants creativity increased (George & Wiley, 2020). Several studies have demonstrated the benefits of diversity of inspiration on creativity: diverse crowd-sourced examples were found to increase creativity in slogan writing (Rhys Cox et al., 2021), and diverse sources of inspiration aided in design ideation (Chan, 2014). Scientific creativity was found to increase after scientists read partially matched articles where a topic was used in an analogically similar but not identical manner to the scientist’s own work, as opposed to fully matched or unmatched articles (Kang et al., 2022).

This boom in research is accompanied by new technologies accessible to writers and creators such as GPT-3 and ChatGPT that are increasingly used by people as ad hoc creativity support tools (e.g., Shidiq, 2023; Stevenson et al., 2022). These new tools are accompanied by an influx of task oriented online products aimed at assisting people with specific writing tasks, such as Sudowrite (www.sudowrite.com) which assists creative writers, Wordtune (www.wordtune.com) which enhances writing clarity, and Jasper (www.jasper.ai) which is aimed at improving marketing copy. Academic work examining the impact of large language models on creative writing includes the work of Lee et al. (2022), which examines the impact of GPT-3-generated text suggestions on author’s story writing process, and finds that writers’ use of the language model varies between writers and use cases. Calderwood et al. (2020) find in an exploratory study that novelists may use language models as antagonists that can help the writer come up with their own ideas, by building off of but ultimately ignoring the model’s suggestions.

However, while many of these works draw on cognitive theories in examining the impact of computer-generated suggestions on creativity or writing, much work remains in developing a greater understanding of the cognitive aspects of human–machine co-creativity (Rafner et al., 2023; Vinchon et al., 2023). Cognitive theories of co-creativity can facilitate the development of new and effective creativity aids, while also contributing to a greater understanding of the human creative process.

### Semantic memory networks and creativity

In this work, we draw on previous research in the cognitive domain and in human–computer interaction to develop a tool that allows investigating the effects of text suggestions on creative ideation in a divergent thinking task. While large language models undoubtedly produce more useful text recommendations, the inability to understand why certain phrases generated by these models are chosen over others makes such systems noisier and more difficult to study from a cognitive perspective. For example, if an LLM was queried to provide help for breaking a creative impasse in an AUT for the object ‘brick’ and it first provides the suggestion ‘use it for something related to gardening’ and then the suggestion ‘build a bookshelf’ there is no natural way to differentiate between the creativity of these two suggestions or to directly quantify their relationship to the participants previous ideas. Here, we draw on research in cognitive network science to develop a creativity support tool which allows us to directly relate the study of creativity support to research regarding the role of semantic memory in creative ideation. The network structure of the model of semantic memory we use allows us some control over the strength of creative associations that we aim to provoke in our tool, as well as allowing us to relate our findings to the existing literature in creativity and semantic memory.

Recent advances in cognitive network science allow applying a quantitative approach to represent the associations between concepts in semantic memory as a semantic memory network (Hills & Kenett, 2022; Siew et al., 2019). This instantiates the metaphor of memory as a cognitive map, on which some sort of mental vehicle travels (Hills & Kenett, 2022). Most theories of memory assume that it is easier to move from a concept to a nearby concept than to a distant one (Kenett et al., 2017; Kumar et al., 2020). The characterization of creativity through semantically distant associations suggests that an ability to navigate between distant concepts in semantic memory leads to greater creativity (Kenett, 2024). Thus, cognitive network science tools provide the possibility of stimulating this and studying it quantitatively. Importantly, the notion of semantic distance as operationalizing semantic similarity has been previously shown in textual corpus-based semantic models, such as WordNet (Ensor et al., 2021). However, such a network-based operationalization is more recent, has been shown to outperform such corpora-based models (Vankrunkelsven et al., 2018; Yang et al., 2023) and generalize across different cognitive domains (Merseal et al., 2023; Siew & Castro, 2023).

Recent work has found that the semantic memory network structure of higher-creative people (typically identified via higher performance on creative tasks, such as the alternative uses task, or self-report questionnaires [see below]) differs from those of less creative people. The semantic memory network of higher-creative individuals is more connected and flexible (Benedek et al., 2023; Kenett, 2024; Kenett & Faust, 2019): meaning that higher-creative individuals associate more distant concepts with each other, while lower-creative individuals tend to only consider semantically near concepts as closely related (Beaty et al., 2021; Gray et al., 2019). This allows higher creative individuals to make creative associations, as they can more easily draw connections between unexpected or distantly related ideas (Beaty & Kenett, 2023). The richer, more flexible semantic memory structure exhibited in higher creativity individuals has been shown to facilitate broader search processes (Kenett, 2024). This evidence – that the way we traverse through memory systems is related to creativity – suggests that semantic memory networks can be utilized to elicit creative ideation. Here, we examine how Divergent Thinking (DT) – the most commonly assessed component of creative thinking (Guilford, 1950), typically measured via the Alternative Uses Task (AUT; Acar & Runco, 2019; Torrance, 1966) – might be enhanced to overcome moments of impasse in the creative process, by sparking associative connections between seemingly distant concepts.

DT has been conceptualized as a semantic search process through memory (Hass, 2017a, 2017b). In contrast to semantic fluency tasks such as naming animals (Ardila et al., 2006), in DT the search process is exploratory and drifts from the topic of interest (such as the cue word in an AUT). Those who can retrieve many concepts through DT are better positioned to associatively combine the concepts into many creative ideas (Silvia et al., 2013). This indicates that distant word recommendations might trigger memory retrieval and broaden one’s search during the AUT by stimulating a new untapped cluster of ideas in semantic memory.

Other theories of creativity such as the Search for Ideas in Associative Memory (SIAM) model, examine the context of a creative idea in relation to the ideas preceding it, or of group members, to explain creative ideation (Nijstad & Stroebe, 2006). The SIAM model suggests that idea generation is a search within associative memory consisting of two stages: knowledge activation, where a concept is retrieved from long-term memory; and idea production, where features of that concept are iterated upon to generate a series of ideas that are closely related. Either stage of the process can fail, leading to a state of impasse. Chan and colleagues have shown that offering participants semantically far stimuli during the knowledge activation phase, and semantically near stimuli during the idea production phase leads to more creative ideas than simply offering far stimuli, validating the SIAM model in practice (Chan et al., 2017; Siangliulue et al., 2015).

In both the associative and SIAM models of creativity, the notion of distance between concepts is central to creative ideation (Kenett, 2019). Research examining semantic distance between words found that words separated by a path length of up to four steps in a semantic memory network are most often rated as related, while words at a higher distance are rated as unrelated to each other. Generally, word-pairs up to three words apart show a stronger priming effect (Kenett et al., 2017; Kumar et al., 2020; Levy et al., 2021). Thus, a quantitative measure of distance between concepts conveys meaningful cognitive information.

The conceptualization of semantic memory as a network allows us to formalize models of search and traversal processes through this network. In this type of model, nodes represent concepts, and edges represent associations or connections between concepts. Once such a network is defined, it is then possible to formalize cognitive processes as network traversal algorithms (Abbott et al., 2012, 2015; Benigni et al., 2021; Bourgin et al., 2014; Kenett & Austerweil, 2016; Marko & Riečanský, 2021; Siew, 2019). For example, creative associative thinking can be modeled as the outcome of a random walk over a semantic memory network with a flexible and highly interconnected structure (derived from higher-creative individuals), while random walks over the semantic memory network structure of lower-creative individuals reach less concepts over the same number of steps in the network (Kenett & Austerweil, 2016). Similarly, the spreadR package (Siew, 2019) which models the long-term result of a random walk on a network effectively models how spreading activation (Collins & Loftus, 1975) on a semantic memory network can account for semantic priming, and effects in false memory and word recognition. We draw on this conceptualization of a semantic memory network on which a random walk process activates different concepts in semantic memory as the basis for our word recommendation tool. By generating word recommendations at various locations in the semantic memory network based on this process, these concepts may be activated, simulating the endpoint of spreading activation through the semantic memory network and triggering new spreading activation processes in semantic areas that can inspire creative associations.

### The present research

The Associative Creativity Sparker (ACS) presented here was developed based on the theory of associative search in memory (Raaijmakers & Shiffrin, 1981), and findings drawing on cognitive network science (Kenett, 2024) to test the associative theory of creativity (Beaty & Kenett, 2023; Mednick, 1962). We propose that when people reach an impasse, word recommendations can potentially pull them out of their fixation by triggering new and creative associations. As such, the aim of the ACS is to spark creative ideation in the impasse state of DT.

We draw on findings that use network science tools to model semantic memory networks, and findings showing that higher-creative individuals form more distant and random associations between concepts than lower-creative individuals. This suggests that the location of word recommendations in semantic memory relative to concepts already active in the participants’ mind may affect the fluency and creativity of subsequent ideas. The ACS allows us to test this hypothesis by generating word recommendations that represent different trajectories through semantic memory in a potential creative thought process. We develop a network traversal method to generate word recommendations that stimulate different associations between concepts and implement this method in an online system. Semantic memory networks can be estimated from empirical aggregated, group-based (De Deyne et al., 2019; Kenett et al., 2011) or individual-based (Benedek et al., 2017; Ovando-Tellez, Kenett et al., 2022b) data (for more discussion, see Siew et al., 2019; Zemla & Austerweil, 2018). However, as individual-based semantic memory networks tend to be smaller and can be less reliable than a group-based semantic memory network (Wulff et al., 2022). Therefore, for our purposes we use a large-scale group-based semantic memory network in English, constructed for 12,000 cue words (De Deyne et al., 2019).

We conduct two studies that demonstrate the use of the ACS. In Study 1, we conduct an initial proof of concept analysis to determine whether providing word recommendations based on different trajectories through a semantic memory network will affect the fluency and originality levels of participants’ responses in the AUT. We varied both the semantic distance of word recommendations from the AUT cue, and the semantic distance between the word recommendations and alternative uses provided by participants before they reach a creative impasse. We predict that word recommendations that are semantically distant from the responses already given by the participant, but sufficiently near the AUT cue, will result in higher fluency and originality. Furthermore, we predict that participants who are more creative will benefit more from distant word suggestions, since their semantic memory network is more diffuse, allowing them to make creative associations between distant concepts. In addition, we examine how participants’ fluid intelligence (*Gf*) and creativity might affect participant’s ability to associate near or far word recommendations into new creative ideas, based on the role of fluid intelligence in creativity (Beaty et al., 2023; Gerwig et al., 2021; Silvia, 2015). We predict that *Gf* and originality will interact with the *Distance* and *Direction* parameters: participants higher in *Gf* and creativity will be better able to associate distant and unrelated concepts into new and creative ideas.

In Study 2, we aim to replicate the effects found in Study 1. In addition, we examine how writing expertise might further affect the utility of co-creative writing support systems, similar to the ACS. We expect that participants with more writing experience will have greater experience generating and adjusting creative writing ideas, and thus will be better able to integrate writing prompts into their idea generation process.

## Study 1

In Study 1, we examine whether the location of word recommendations in a semantic memory network relative to the pre-impasse responses in the AUT affects (1) the ability of these word recommendations to break participants out of creative fixations and (2) the originality of the responses they give after the word recommendations. This is achieved via the Associative Creativity Sparker (ACS), a novel tool we developed to break people from creative block. We also examine the effects of fluid intelligence (*Gf*), overall creativity and literary creativity on the participants’ ability to break out of fixations with the ACS.

### Materials and methods

#### Participants

One hundred and twenty-one participants were recruited through Prolific (mean age = 37.4 years, SD = 13.3 years, 74 female) to participate in this study. Sample size was based on previous studies which assessed AUT and *Gf* (Beaty et al., 2023; Beaty & Silvia, 2012; Gilhooly et al., 2007). Participants were from the UK (66), the US (20), Canada (11), and Ireland (10), and the median participant had a bachelors’ degree. One hundred and seven participants were included in the final analysis. Fourteen participants were excluded due to failed attention checks, technical issues, age above 70 or failure to complete the tasks as instructed. Participants were compensated 4.5 pounds, participated voluntarily and provided informed consent. The study took 35 min to complete on average and was approved by the Technion - Israel Institute of Technology Institutional Review Board.

### Methods

#### The Alternative Uses Task (AUT)

The AUT is a DT task used to measure creative idea generation (Acar & Runco, 2019). The AUT is commonly used to measure the fluency and originality of ideas (Beaty et al., 2022). Participants were required to generate alternative uses to five objects (broom, knife, lamp, shoe, and towel), and had unlimited time to generate any creative alternative uses they could think of for each of these items. These items were selected from Beaty et al. (2022) because they were shown to produce similar numbers of AUT responses. The order of the AUT objects was randomized. Participants were instructed to respond creatively to the task, as this has been shown to influence the creativity level of responses (Said-Metwaly et al., 2020). The specific instructions given where:

You will be asked to produce as many creative uses as you can think of, which are different from the normal use, for five common objects. For example: the common use for a newspaper is reading, but it could also be used for "swatting flies", "to line drawers", "to make a paper hat" and so on. Please try to produce possible uses which are different from the normal one and different in kind from each other. The creative alternative uses should be short phrases. When you run out of ideas, you should click on get recommendation, which will show you a word that might help you find new ideas. You can then add and submit these new ideas. Once you run out of ideas again, click on ‘I am completely out of ideas.’

In addition, there was an interactive demo showing how to use the system before the first ACS trial.

#### The Associative Creativity Sparker (ACS)

We modified the AUT, such that when participants reached an impasse state and indicated they ran out of ideas, they were required to ask for a word recommendation and after its presentation, were asked to provide any new alternative uses they had. These word recommendations were automatically generated by the ACS. Participants were required to provide at least one response in the first part of each AUT trial but were not required to provide any response after the word recommendation. The ACS consists of the following components: (1) the website, (2) the free association-based semantic memory network, and (3) the word recommendation algorithm. The ACS is built modularly, so that it is easy to replace the existing word recommendation algorithm with any other desired method for word or phrase recommendations in the AUT. These can also be semantic network based, but it is equally easy to add in any other method for word or phrase recommendations such as those produced by generative AI. The ACS code can be found at https://github.com/TaliaWise/ACS/tree/main.

##### The website

**Fig. 1:**
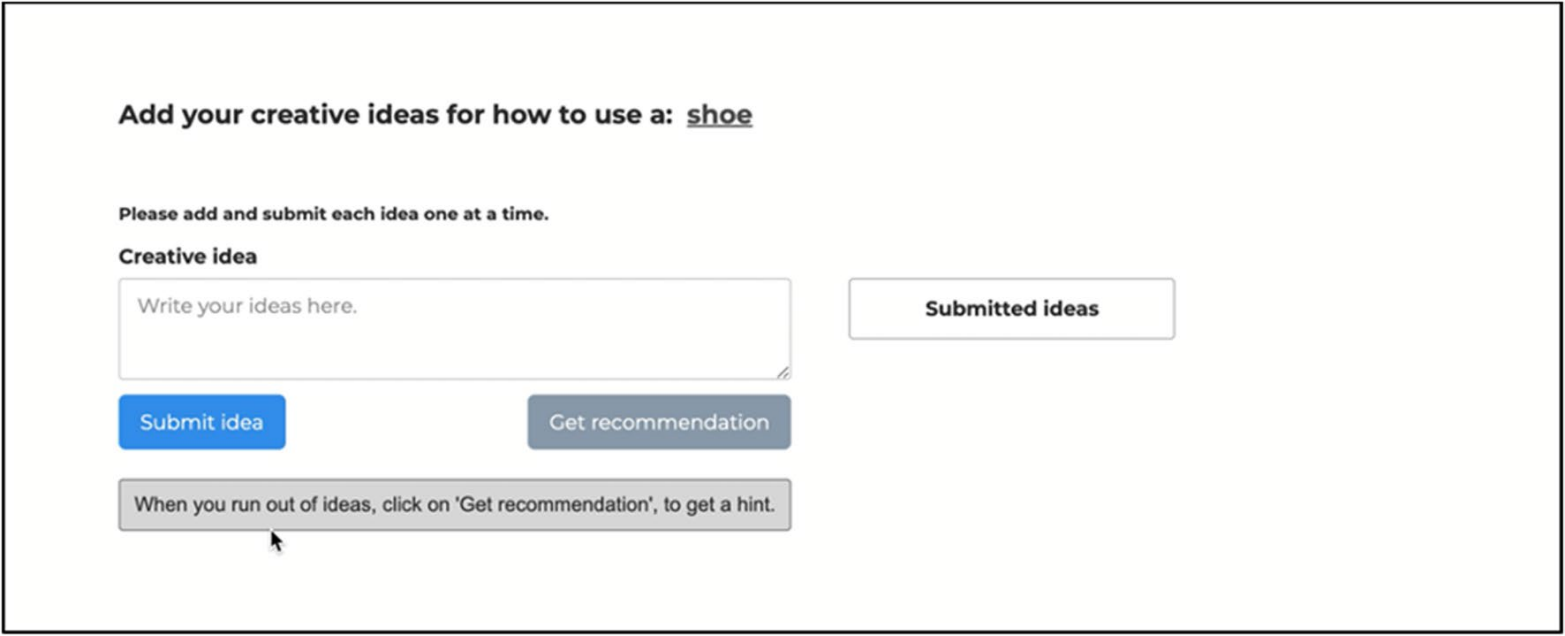
The Associative Creative Sparker Web-app. In this instance, the participant is instructed to type in creative alternative uses for a shoe. The participant can type in as many uses as they wish, and these uses appear on the right of the screen under ‘Submitted ideas.’ Once the participant reaches an impasse, they request and are shown a word recommendation. The participant can then add and submit any new creative ideas they have

We developed the ACS as a flask web app, which runs on Heroku servers using a Postgresql database. The app is developed to be modular, allowing for easy adjustment of experiment parameters, including the number of words recommended, the randomization of experiment conditions, and the number and names of AUT objects. In addition, the modular design of the ACS allows for relatively easy changes to the word recommendation algorithm, which could facilitate changes such as integrating AI models into the recommendation system. The ACS is accessible online anywhere. It is designed to allow participants to enter responses to a task and generate word recommendations in real time that are personalized to their previous responses (Fig. 1).

##### Free association-based semantic memory network

We used the English “Small World of Words” free association dataset (De Deyne et al., 2019), to generate a semantic memory network. This dataset consists of word associations for over 12,000 cue words and participants were required to provide three responses for a subset of 14 cue words (De Deyne et al., 2019). This semantic memory network is used as the scaffolding of the ACS, which realizes a search process over it – loosely based on a random walk algorithm (Lawler & Limic, 2010) – to retrieve cue words from the network with varying distances to the originating AUT object.

The word recommendation algorithm described below crawls this network to generate word recommendations at different locations in semantic memory compared to the AUT cue and the participants’ previous responses. Our network consists of 11,512 nodes (English words) and 36,383 edges (free-association connections between the words). The nodes are derived from the list of cue words and participant generated word-associations from (De Deyne et al., 2019), and we create an edge in the network connecting two nodes if at least ten people freely associate this pair of words in the dataset. Our semantic memory network contains only the largest connected component of the dataset.

##### Word recommendation algorithm

We developed a word recommendation algorithm that generates words based on their *distance* to the AUT object in the semantic memory network (1 to 4 nodes away from the object in the network), and their *direction* towards or away from the previous responses to the AUT. For each AUT object a random *distance* and *direction* are chosen. The *direction* of word recommendations describes how close the word is to the participant’s pre-impasse ideas in the ACS trial. Towards recommendations have a low average path length in the semantic network to the pre-impasse responses, and Away recommendations have a high average path length in the semantic network to the pre-impasse responses.

Participants responses are processed through an online text analysis pipeline to compute the direction parameter. First, responses are made lower case, cleaned of punctuation, and cleaned of extra white space using the python string package. The response phrases are then separated into individual words, and only words which are not stop-words (words such as prepositions and conjunctions which are unimportant to the task) are included in the final list, according to a ‘bag of words’ set-up. The list of stop-words was derived from the NLTK natural language processing toolkit (Bird, 2006). This pre-processing pipeline loosely follows that used in the SemDis platform (Beaty & Johnson, 2021). The list of words is then assumed to represent the ‘idea space representation’ of participant’s previous responses in the trial. To compute the direction of word recommendations, only the words in the ‘idea space representation’ which exist in the semantic memory network are used, since words not in the network have undefined path length.

**Fig. 2:**
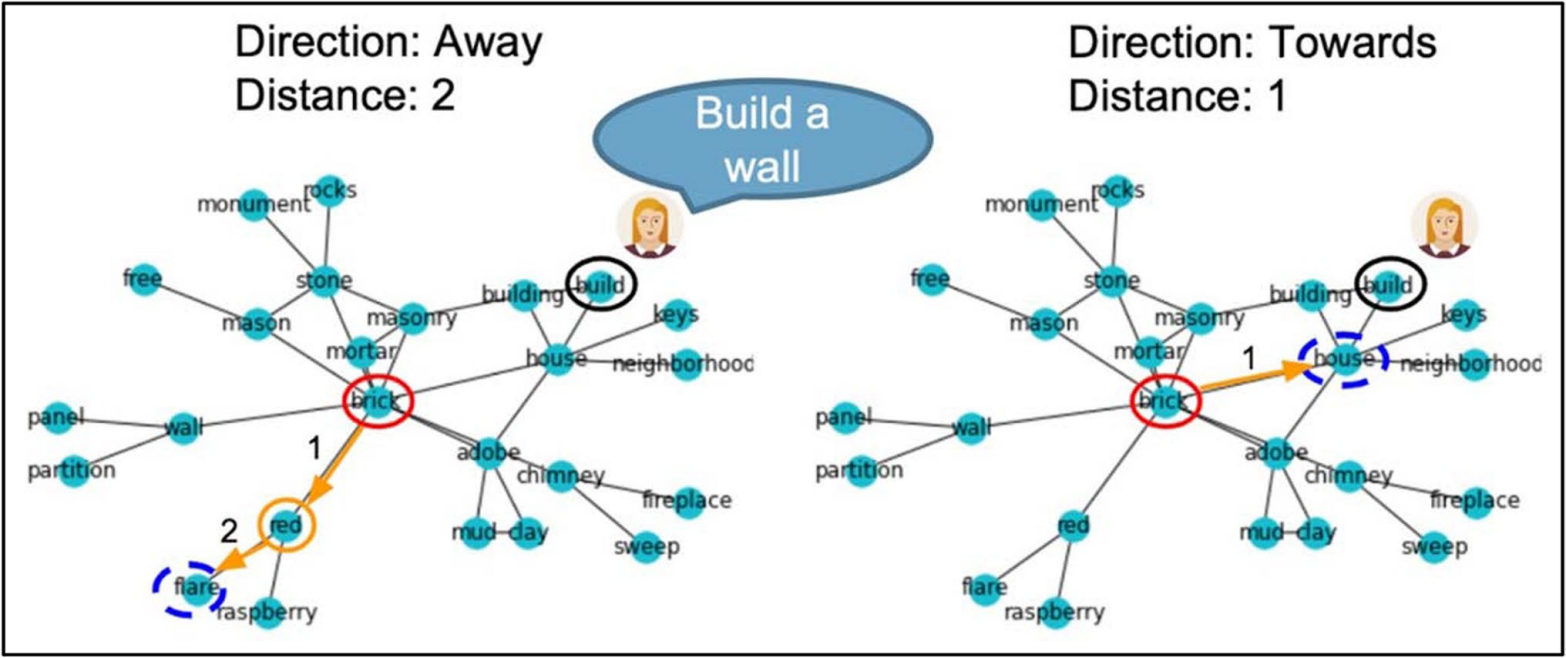
The word recommendation algorithm. In this example, the participant is asked to generate creative alternative uses for a brick. The (hypothetical) participant produces the response ‘build a wall’ and reaches an impasse – fixating on the typical use of bricks. Two possible word recommendation scenarios are shown. *Left*: A word recommendation that is at a distance of two steps from brick in the semantic network and directed away from the words in the participants previous response, generating the word ‘flare.’ *Right*: A word recommendation at a distance of one step away from brick and directed towards the words in the participants previous response, generating the word ‘house’. One can imagine how if the word ‘flare’ successfully inspires a new use of a brick, this use would be more creative and unusual than a use sparked by the word recommendation ‘house’

To select the word to be recommended all nodes at the chosen distance from the object node in the semantic memory network are sorted according to their average *distance* from the ‘idea space representation’ list of the participant’s pre-impasse responses to the trial. To get a word recommendation that is *towards* the previous responses, the word with the lowest average distance to the pre-impasse ‘idea space’ is selected, resulting in a closely related word recommendation to the ideas the participant already had. For an *away* recommendation, the average network distance to the pre-impasse ‘idea space’ is maximized, resulting in a novel word recommendation relative to the ideas the participant already had. If the selected word recommendation was already used in a response by the participant, the next best word is selected and shown to the participant instead (Examples of word recommendations are illustrated in Fig. 2).

#### ACS output measures

The ACS measures multiple variables related to participants’ performance in it:

Fluency of responses is measured through the number of responses participants provide. The ACS has two measures of fluency, *fluency before impasse* measures the number of responses the participant had before running out of ideas, corresponding to the typical fluency measure in an AUT. *Fluency after impasse* measures the number of ideas after the word recommendation is seen, this represents the added value to participant fluency provided by the word recommendation.

Likelihood of impasse break is a binary outcome variable which measures if a word recommendation successfully sparked new ideas after the participant reached impasse, by measuring if a trial has at least one response after impasse or none. This measure is 1 if the participant had at least one response after the word recommendation, and 0 if the participant had no responses after the word recommendation. In contrast to a typical AUT, in the ACS participants are already in a state of creative fixation, and thus often do not have any ideas at all, making this variable different from a typical fluency measure. The likelihood of impasse break represents whether the word recommendation succeeded at breaking the participant out of an impasse state in a given trial.

Originality of responses is measured through human subjective originality ratings (Kaufman, 2019; Silvia et al., 2008), and not quantitative originality measures (Beaty & Johnson, 2021; Dumas et al., 2021; Organisciak et al., 2023). This is because such quantitative measures are suited for aggregated responses, or latent factors, whereas in our study we focused on response level effects of the ACS. For our subjective scoring, we follow a common scoring scale ranging from 1 (not at all original) to 5 (very original) scale (Hass et al., 2018).

Two graduate students rated the ACS responses. In addition, the responses were subdivided into groups of 200–300 and rated by 3–7 crowd-workers recruited from Prolific. The ratings from the two graduate students and the crowd-workers were aggregated and an ICC(1,k) score was computed for each group of ratings (Shrout & Fleiss, 1979). Because several crowd-workers submitted arbitrary seeming responses (e.g., repeated patterns of ratings), the ratings from any crowd-workers who significantly reduced the ICC(1,k) score (and thus who seem to have provided arbitrary ratings) were removed, resulting in a final ICC(1,k) score of over 0.7 for each group of ratings. The ratings of the graduate students and remaining crowd-workers were then averaged to produce a final originality score for each response. We measure originality of the responses both before and after the word recommendations, resulting in *pre-impasse originality and post-recommendation originality measures for each trial. This allows us to analyze the impact of word recommendations on response originality and consider whether post-recommendation responses are* more or less original than pre-impasse responses, and whether response originality differs between word-recommendation conditions.

Response time (RT) is measured as the time between the beginning of each response (which can be the start of a trial, end of previous response, or the time the word recommendation is shown) until the participant begins typing each response. This represents the time spent thinking before writing each idea. This is an outcome variable that can validate that participants reached a state of creative fixation; we expect that as they approach an impasse state the time before each response will increase. For the responses after impasse, we expect that the time spent thinking will vary between word recommendation types, as ‘aha’- type creative associations could have a different thinking time than more deliberately derived ideas.

##### Additional measures

The ACS also includes other measures that were not used in the current studies. These include key-log timing, enabling researchers to identify the exact moment that a response or word was typed, or submitted to the system, and a measure of response adherence, showing how semantically close the response was to the word recommendation.

#### Creativity assessment

Participants’ creativity was assessed via the Inventory of Creative Achievement and Activity (ICAA) questionnaire (Diedrich et al., 2018), applied to assess individual differences in creativity. The ICAA measures creative activity and achievement in eight different domains: music, literature, sports, science, cooking, visual arts, performance arts and handicraft. For each domain participants are asked to note their personal and professional achievements, and the number of times in the last 10 years they have pursued activities in that domain. Because the activity and achievement scores are highly correlated, we used principal component analysis to compute a composite score for each participant. We computed PCA eigenvalues and factor loadings to determine that a unidimensional model fits the data (Forthmann et al., 2023; Gilmer & Feldt, 1983). The eigenvalue for this model was 0.829, and the factor loadings for the two variables were 0.70 and 0.70 respectively, showing that the final composite score had a strong correlation to each variable. The factor determinacy index (Ferrando & Lorenzo-Seva, 2018) on the final extracted factor was found to be 1.67.

Furthermore, activities and achievements in the domain of literature were analyzed individually in addition to the overall achievement and activity scores, as we expected creativity in this domain to be especially relevant to the task. A composite literature experience score is computed using principal component analysis on the literature activity and literature achievement scores. To determine that a unidimensional model fit this data we computed the factor loadings [0.71, 0.71] and eigenvalue (0.672), showing that the PCA factor captured a large proportion of each variable’s variance (Forthmann et al., 2023; Gilmer & Feldt, 1983). The factor determinacy index (Ferrando & Lorenzo-Seva, 2018) computed on the extracted factor was found to be 1.78.

#### Fluid intelligence assessment

Fluid intelligence (*Gf*) was measured based on Kenett, Beaty et al. (2016a) via three tasks: (1) a series task from the Culture Fair Intelligence Test (CFIT), which involves choosing an image that completes a series of images (13 items, 3 min; Carroll, 1993); (2) A letter-sets task, which presents a series of four-letter combinations and requires people to determine which set does not follow a rule governing the other four (16 items, 4 min; Ekstrom et al., 1976); and (3) A number-series task, which presents a sequence of numbers and requires participants to discover a rule governing their change (15 items, 4.5 min; Thurstone, 1938). To compute a general composite *Gf* score, we use principal component analysis, by summing the multiplication of each independent *Gf* score by its weight of the first unrotated principal component (Kenett, Beaty, et al., 2016a). To determine that a unidimensional model fit this data we computed the factor loadings [0.5051805, 0.59497742, 0.62513561] and eigenvalue (0.588), showing that the PCA factor captured a large proportion of each variable’s variance (Forthmann et al., 2023; Gilmer & Feldt, 1983). The factor determinacy index (Ferrando & Lorenzo-Seva, 2018) computed on the extracted factor was found to be 1.78.

#### Statistical analysis

Impasse break and originality outcome variables were analyzed using linear mixed-effects (LME) hierarchical regression models (Baayen et al., 2008), as implemented in the lme4 package (Bates et al., 2015) in R. Because each participant completes the ACS four times with a random distance and direction parameters for each trial, LME models were the most suitable method for analyzing the difference in outcomes between the various groups of distance and direction. This approach was selected for its ability to handle the substantial variation in individual responses to word recommendation conditions. It permits an in-depth examination of the primary variables of interest by incorporating a random intercept for each participant, thereby accounting for inter-individual differences. In this analysis, a random intercept for each participant removes some of the influence of the variability between individuals on the final fixed-effects comparison. The model also accounts for variability due to demographic factors; age, sex, country, education, circumstantial factors; experiment order, AUT object, idea order (the order of responses in the trial, normalized so that 1 is always the first response after impasse and all responses before impasse count back from 0, responses after impasse count forward from (1), and individual factors; *Gf*, and creative activities and achievements. These factors are assessed before including the effects of the word recommendations on the outcome variables. This ensures that any potential effect of the word recommendations on the outcome is above and beyond the effects of the individual and group variability. The final estimate is thus a more generalizable estimate of the true effect of the fixed effects of interest (distance, direction, *Gf,* and creativity scores) on the outcomes (impasse break and originality of responses).

To assess the impact of word recommendations on originality, the Distance and Direction word recommendation parameters are coded as before or after the word recommendation, thus allowing the LME model to compare each word recommendation condition to the pre-impasse responses. The pre-impasse responses thus serve as a control condition.

First, we analyzed ACS impasse break. For this analysis we aggregated responses by trial, where each trial has a binary outcome of whether the participant had ideas or not after seeing the word recommendation. We build a linear mixed effects model based on the response after impasse outcome variable, so that the model computes the likelihood of response for each category of word recommendation. We then analyze the originality of responses. This model is run on the disaggregated individual responses.

In both models we used a bottom-up process, where we start with a baseline model which only has the participants’ ID as a random effect, and iteratively add each variable of interest and test for statistical significance. The first variable is added to the baseline model and we test if the addition of this variable significantly improves model fit compared to the baseline. If the new variable improves model fit it is kept as the new ‘best’ model and the next variable is added to it and compared to it. If adding the variable did not improve model fit, the next variable is added to the baseline and compared to the baseline. This process is further illustrated in Tables 2, 3, 4 and 5. For each analysis, we first tested the impact of demographic variables (age, sex, country, and years of education) but these did not significantly improve model fit for any of the outcomes, so we do not include them in the final analysis. We then tested the effects of *Gf*, creative activity and achievement and literature activity and achievement. For the aggregated fluency model, we then added the fluency before impasse measure, and found that it did not significantly improve model fit. For the originality model, which was not aggregated, we included idea order which significantly improved model fit. We then tested our hypothesized effects of distance, direction and distance x direction for both models.

#### Procedure

Participants completed the ACS for five different objects. This included an initial instruction page describing the interface and explaining how to use the task, followed by an interactive instruction guide for using the ACS interface. The five trials were then given in sequence. For each trial, participants first provide unassisted creative uses for the object until they reach an impasse, then request word recommendations, and finally may input any new ideas they have. Following this, participants answered the ICAA, *Gf*, and demographic questionnaires, which were administered on Qualtrics. Each questionnaire had an instruction page followed by the question page. Participants had the option of taking short breaks between questionnaires. Attention checks were measured during the ICAA, randomly tested between the three *Gf* tasks, and in the demographics section.

### Results

We examined all responses generated by participants (see Table 1 for examples). We then evaluate the predictors of impasse break and originality.

**Table 1:** Examples of participant responses

Cue-object	Parameters	Recommendation	Responses
Towel	Distance: 1		To wear as a dress
	Direction: *Towards*		To do the ironing on if you don't have a board
			To cut up and use for smaller towels
			To cut up and use to make something like patch
		Beach	
			To use on the beach
			To use as a picnic blanket
Lamp	Distance: 3		Use the shade as a mad hat
	Direction: *Away*		Use it in a cupboard
		Hopes	
			Holding it above a child's play theatre

In the top example the participant is asked to generate creative uses for a towel, and fixates on uses related to clothing (dress, ironing, patch). Once at an impasse, the participant is shown the word-recommendation ‘Beach’ which is in condition distance:1, direction: towards – the closest possible word recommendation in our system. This leads to the highly common uses of using the towel on the beach or as a picnic blanket. In the bottom example, the participant generates two fairly common but different uses for a lamp: as a hat or to light a cupboard. The participant is shown the word recommendation ‘Hopes’ which is quite far (distance 3, direction: away). The participant then gives a more creative use ‘Holding it above a child’s play theater’

Impasse break occurred in 33% of the ACS trials and the word recommendations were helpful in at least one trial for 78% of participants (Table 1 shows two trials with successful impasse break). To analyze what factors affected impasse break, participants AUT responses were aggregated by trial, so that each trial had as an outcome a binary measure of having one or more responses after the word recommendation (impasse break) or none (participant remains in the impasse). Two individual AUT trials were excluded due to technical issues, although the remaining four trials of each of these two participants were still used, and 14 of the participants completed three trials instead of five, leaving (*N* = 505) trials included in the analysis.

For this model, participants ID and trial order were both included as random variables as the baseline model. We used a bottom-up model selection mechanism where each variable was added and compared to the preceding significant model. If adding the new variable resulted in a model that was significantly different from the previous best model, the new variable was kept, and this new model is now considered the best model. Table 2 shows this analysis. We started with the baseline model and first analyzed the addition of demographic variables age, sex, years of education and country as random effects. None of these significantly improved model fit over the baseline, so these were not included in the final analysis. Trial object was then analyzed as a random effect but did not improve the model fit over baseline. We then analyzed the fixed effect of *Gf* on impasse break. *Gf* significantly improved model fit over the baseline, so further models were compared to this one. Following this we analyzed the fixed effects of creative activities and achievements, literature activities and achievements, and participants’ number of responses (fluency) before impasse on the impasse break outcome measure. None of these variables significantly improved model fit over the *Gf* model. Finally, we analyzed the fixed effects of the distance and direction of word recommendations on impasse break, and the interaction of distance and direction. Both distance and distance $\times$ direction had nearly significant effects. We also tested the interaction of distance $\times$ direction $\times$ *Gf,* but this too did not significantly improve model fit. The final model had the baseline random effects of subject ID and trial order and showed a positive effect of *Gf* on impasse break Est = 0.049, SE = 0.020, *p* = .015.

**Table 2:** Chi-square difference tests for *impasse break*

Model parameters	Log-likelihood	AIC	${\chi }^{2}$	*df*	*p* value
Baseline	– 302.46	612.92	NA	NA	
Trial object	– 302.40	614.80	0.12	1	.727
*Gf*	– 299.46	608.93	5.98	1	.014
*Gf* + Creativity (C)	– 298.91	609.81	1.11	1	.290
*Gf* + Lit	– 299.05	610.11	0.82	1	.364
*Gf* + nIdeas	– 299.44	610.87	0.06	1	.806
*Gf* + Distance	– 297.92	607.83	3.09	1	.078
*Gf* + Direction	– 299.45	610.90	0.03	1	.869
*Gf* + Distance * Direction	– 295.99	607.97	6.95	3	.073
Gf * Distance * Direction	– 294.62	611.23	9.69	6	.138
Baseline model: *Impasse break ~ 1 + (1| subject ID) + (1 | Trial order)*				${R}_{m}^{2}$	${R}_{c}^{2}$
Final model:* Impasse break ~ Gf + (1| subject ID) + (1 | Trial order)*			Final Model	.019	.158

For the final model, we report the marginal and conditional *R*^2^ values (Nakagawa & Schielzeth, 2013), which show the variance explained by the fixed effects (marginal), and entire model including both random and fixed effects (conditional) respectively

Next, the effect of word recommendations on response originality was analyzed similarly to impasse break. For this model, each response was analyzed individually. Only trials which had at least one post-impasse response were included in this analysis, leaving 310 trials from 102 participants. From the 310 trials included in the analysis, we include 599 post-word recommendation responses, compared to 1055 pre-impasse responses which serve as a control. For this model, only subject ID was included as a random effect, because the trial order intercept was found to have a variance and standard deviation of zero. Similarly to the impasse break model, the model was analyzed in a bottom-up manner where variables were added one at a time and compared to the previous best model (Table 3). If the addition of a variable resulted in a significantly better model, the ‘previous best’ model was updated to include this variable. We began by analyzing the effect of demographic variables (age, sex, country, education) as random effects. None of these had significant effects of model fit so they are not included in the final analysis (Table 3). We then analyzed trial order and trial object compared to the baseline. The addition of trial object resulted in a significantly better model than the baseline, so this model became the comparison model. Next, we analyzed the effects of *Gf,* creative activities and achievements, and literature activities and achievements, none of which significantly improved model fit. Idea order was then tested and found to improve model fit. Finally, we analyzed the effects of word recommendation distance and direction, distance $\times$ direction and compared these to the previous best model. Specifically, we test the effects of close (1–2 steps) and far (3–4 steps) compared to the originality of pre-impasse (hereinafter control) responses and test the effects of towards and away vs. the originality of control responses. Distance and the interaction of distance and direction were found to significantly improve model fit. We then tested the effects of distance $\times$ direction $\times$ creativity (compared to the distance $\times$ direction model), as this tests our a priori hypothesis, but did not find this improved model fit.

**Table 3:** Chi-square difference tests for *originality*

Model parameters	Log-likelihood	AIC	${\chi }^{2}$	*df*	*p* value
Baseline	– 2289.9	4585.8	NA	NA	
Trial order	– 2289.9	4587.8	0.002	1	.964
Trial object (T)	– 2256.1	4520.2	67.59	1	< .001
T + *Gf*	– 2255.3	4520.7	1.51	1	.218
T + C	– 2255.3	4520.6	1.59	1	.206
T + Lit	– 2255.7	4521.5	0.70	1	.401
T + Idea Order (IO)	– 2217.3	4444.6	77.60	1	< .001
T + IO + Distance	– 2211.9	4437.8	10.75	2	.004
T + IO + Direction	– 2215.7	4445.5	16.32	2	.210
T + IO + Distance * Direction	– 2209.9	4437.8	14.48	4	.005
T + IO + Distance * Direction * C	– 2207.7	4443.4	4.42	5	.489
Baseline model: *Originality ~ 1 + (1| subject ID)*				${R}_{m}^{2}$	${R}_{c}^{2}$
Final model: Originality ~ (1|T) + IO + Distance * Direction + (1| subject ID)			Final Model:	.042	.267

For the final model, we report the marginal and conditional *R*^2^ values (Nakagawa & Schielzeth, 2013), which show the variance explained by the fixed effects (marginal), and entire model including both random and fixed effects (conditional) respectively

**Fig. 3:**
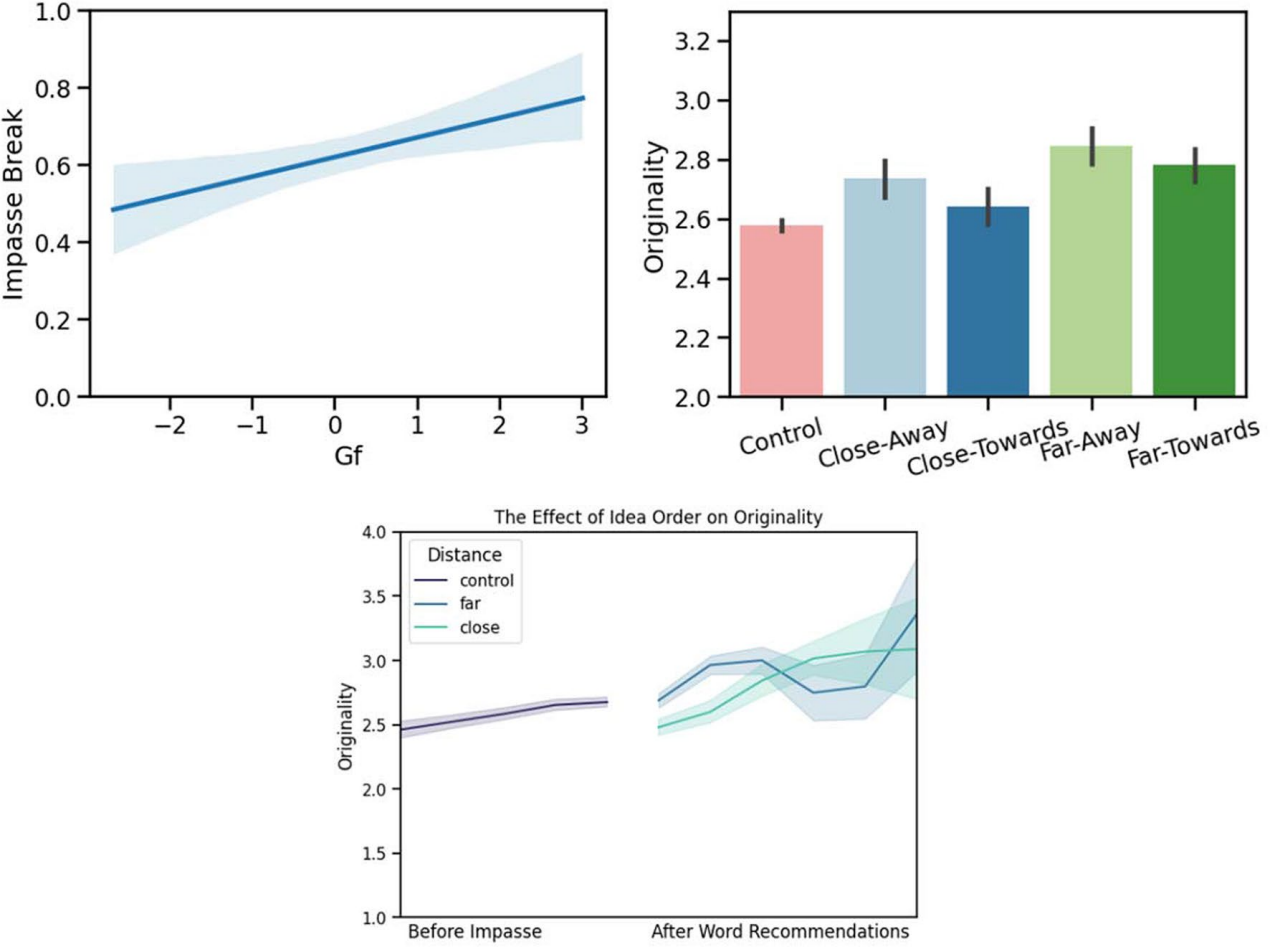
ACS impact on impasse break in Study 1. *Top left*: The impact of *Gf* on impasse break. *Top right*: The impact of word-recommendations on originality. *Bottom*: The effect of idea order and word recommendation distance on originality

We examined the coefficients and estimations of each fixed effect in our final model on the outcome variable *originality of responses*. In the final model (Table 3), the random effects were subject ID, Var = 0.107, SD = 0.327, and trial object (T), Var = 0.022, SD = 0.148. The final model had significant effects of idea order, Est = .165, SE = .020, *p* < .001, and distance $\times$ direction. For this variable we find that close-towards recommendations lead to responses significantly less original than control (pre-impasse) responses, Est = – 0.217, SE = 0.064, *p* < .001. When comparing the different word recommendation conditions, we also find that the close-towards recommendations resulted in responses significantly less original than the other three types. The close-away responses were Est = 0.154, SE = 0.007, *p* = .044, more original than close-towards, far-towards were Est = 0.25, SE = 0.079, *p* < .001, more original, and far-away were Est = 0.247, SE = 0.0806, *p* = .002 more original (Fig. 3).

### Discussion

Study 1 demonstrates that the ACS is effective at breaking participants out of creative fixations. The word recommendations inspired new ideas in 33% of the trials and were helpful for 78% of participants in at least one trial. This study also demonstrates that the distance of the word-recommendations relative to the cue object and direction relative to previous responses affect participants’ originality of responses. Responses following word recommendations that are far from the cue object are more original than close word recommendations. However, this effect is moderated by the direction of word recommendations: responses that are close to the object cue and towards the participant’s previous ideas are the least original of all, both compared to pre-impasse responses and the other types of word recommendations. In Study 1 we find that participants’ fluid intelligence (*Gf)* affects impasse break, but that creativity and literature experience as measured through the ICAA do not affect any outcome variable.

## Study 2

In Study 2, we sought to replicate and extend the results from Study 1 using a larger sample of participants and by focusing on expertise in the literature domain. Additionally, because our study focuses on breaking creative blocks of which writer’s block is a classic example, in Study 2 we examine how novice and experienced writers differ in their ability to benefit from word recommendations in the ACS. We hypothesize that participants with more writing experience will have greater experience generating and adjusting creative writing ideas, and thus will be better able to integrate writing prompts creatively than novice writers.

### Materials and methods

#### Participants

To identify the groups of novice and expert writers, we conducted a preliminary survey collecting 500 participants’ levels of activity and achievement in the domain of literature. We used the literature section of the ICAA for this survey and added several more questions about writing experience as sanity checks (see Appendix). After excluding 60 participants who failed attention checks, provided incomplete responses, or indicated they did not want to take part in the follow-up study, we analyzed the survey responses to determine the novice and experienced writer groups. Responses to the additional sanity check questions closely correlated with participants stated activities and achievements in literature. As such, we applied a PCA on the activity and achievement scores to compute a single literary creativity score for each participant. To be consistent with sample size of Study 1, the top scoring 120 participants were labeled as experienced writers and bottom 120 participants labeled as novice writers.

These 240 participants from the initial filtering questionnaire on Prolific were invited to return and complete the study. Of these, 204 completed the study without technical issues and were included in this analysis. A total of 104 are experienced writers (mean age = 41.34 years, SD = 11.03 years, 71 female), and 100 are novice writers (mean age = 35.26 years, SD = 10.50 years, 59 female). The two groups exhibited significant differences in the stated writing experience of these two groups: Novice writers had (M = 0.69, SD = 3.22) years of writing experience, and expert writers wrote for a mean of 12.39 years, SD = 9.62, *t*(203)= – 11.59, *p* < .001.

All participants are from the UK and native English speakers. All participants adequately completed the questionnaires and attention checks, so all participants’ responses were included. Five individual trials were excluded from the analysis due to technical issues. Participants were compensated 0.15 pounds for the 2-min preliminary survey, participated voluntarily and provided informed consent. Participants who completed the main study were compensated a further 4.5 pounds. The study took 35 min to complete on average. This study was approved by the Technion – Israel Institute of Technology Institutional Review Board.

### Materials

#### ACS

The same website as in Study 1 is used to run the ACS trials, using the same text analysis and word recommendation method. A new Heroku database is used to store results.

#### ACS Measures

As in Study 1, we measure impasse break: whether the trial has at least one response or not and response originality.

#### Creativity assessment

As in Study 1. We computed a single factor using PCA on the ICAA activities and achievements scores. To determine that a unidimensional model fit this data we computed the factor loadings [0.707, 0.707] and eigenvalue (0.860), showing that the PCA factor captured a large proportion of each variable’s variance (Forthmann et al., 2023; Gilmer & Feldt, 1983). The factor determinacy index (Ferrando & Lorenzo-Seva, 2018) computed on the extracted factor was found to be 1.73.

In addition to the literature experience (novice/expert) category we extracted from the initial participant selection process, we computed a factor score for literature activities and achievements from the ICAA to be consistent with Study 1. To determine that a unidimensional model fit this data we computed the factor loadings [0.707, 0.707] and eigenvalue (0.771), showing that the PCA factor captured a large proportion of each variable’s variance (Forthmann et al., 2023; Gilmer & Feldt, 1983). The factor determinacy index (Ferrando & Lorenzo-Seva, 2018) computed on the extracted factor was found to be 2.16.

#### Fluid intelligence assessment

As in Study 1, we computed a single factor using PCA on the three *Gf* tests. To determine that a unidimensional model fit this data we computed the factor loadings [0.540, 0.614, 0.574] and eigenvalue (0.593), showing that the PCA factor captured a large proportion of each variable’s variance (Forthmann et al., 2023; Gilmer & Feldt, 1983). The factor determinacy index (Ferrando & Lorenzo-Seva, 2018) computed on the extracted factor was found to be 1.79.

#### Measures

Following Study 1, we assess *Gf* via the CFIT, letter-sets task and number-series task. We use principal component analysis to compute the general *Gf* score (Kenett, Beaty, et al., 2016a). To measure participants’ creativity, we use the ICAA, and compute a composite score using principal component analysis on the activity and achievement scores. Participants’ writing experience is derived from the initial writing expertise group selection using the literature activity and achievement section of the ICAA. In addition, we added several questions about participant writing experience and experience of writer’s block in their everyday life and in the ACS (These questions are listed in Appendix).

#### Statistical analysis

As in Study 1, we analyze the fixed effects of interest (participant literature experience, *Gf*, creativity, distance, and direction) on the outcomes (impasse break and originality of responses) using linear mixed effects models. We also test the effects of age, sex, years of education, and trial order, and include the random effect of participants ID in all models.

#### Procedure

This study repeated the experimental set up of Study 1. Participants completed the ACS for five different objects, in the same way as in Study 1, as well as completing the same *Gf* and ICAA questionnaires. In addition, in this study we added a questionnaire following the trials which examined participants’ subjective experience of writing with the help of word recommendations. The questions are listed in appendix A. After the five ACS trials and the writing questionnaire participants answered the ICAA, *Gf*, and demographic questionnaires, with accompanying attention checks as in Study 1.

### Results

We examined all responses generated by participants for clarity, and then analyzed the impact of word recommendations on fluency, originality, and response time. Finally, we analyzed participants’ subjective experience of writer’s block in the ACS.

We begin by analyzing the impact of word recommendations on impasse break. Participants had at least one response to the word recommendations in 64% of trials, and 95% had a response after the word recommendation at least once. As in Study 1, responses were aggregated by trial and we model likelihood of *impasse break*, as outcome variable. A total of 1008 trials were included in the analysis, of which 648 have at least one response after impasse. We conduct the same analysis as in Study 1 with the addition of writing experience as an independent variable (Table 4).

**Table 4:** Chi-square difference tests for* impasse break*

Model parameters	Log-likelihood	AIC	${\chi }^{2}$	*df*	*p* value
Baseline	– 602.60	1215.1	NA	NA	
Trial object	– 601.95	1219.9	1.29	4	.862
*Gf*	– 601.55	1213.1	3.99	1	.045
*Gf* + Lit	– 596.01	1204.0	11.09	1	< .001
*Gf* + Lit + C	– 593.86	1201.7	4.29	1	.038
*Gf* + Lit + C + nIdeas	– 593.62	1203.2	0.46	1	.494
*Gf* + Lit + C + Distance	– 592.97	1201.9	1.78	1	.181
*Gf* + Lit + C + Direction	– 590.68	1197.4	6.35	1	.011
*Gf* + Lit + C + Distance * Direction	– 588.94	1197.9	9.83	3	.019
Gf * Distance * Direction + Lit + C	– 587.86	1201.7	2.15	3	.541
*Gf + C +* Distance * Direction * Lit	– 585.34	1196.7	7.20	3	.065
*Gf +* Lit *+* Distance * Direction * C	– 588.23	1202.5	1.41	3	.701
Baseline model: *Impasse break ~ 1 + (1| subject ID) + (1 | Trial order)*				${R}_{m}^{2}$	${R}_{c}^{2}$
Final model: Impasse break ~ Gf + Lit + C + Distance * Direction + (1| subject ID) + (1 | Trial order)			Final Model	.041	.182

For the final model, we report the marginal and conditional *R*^2^ values (Nakagawa & Schielzeth, 2013), which show the variance explained by the fixed effects (marginal), and entire model including both random and fixed effects (conditional) respectively

For impasse break, we began with a baseline model including participants ID and trial order as random effects. We then tested the effects of the inclusion of demographic variables (age, sex, education) as random effects and compared each of these to the baseline model. None significantly improved model fit so they were not included in the final analysis. We then tested the effect of the inclusion of trial object compared to the baseline, but it did not improve model fit, so was not included in later models. Next, we tested fixed effects. We first tested *Gf* which like in Study 1, significantly improved model fit over the baseline, so was included in later models. In Study 2 we also found that the model with *Gf* + Lit was significantly better than *Gf* and *Gf* + Lit + creativity was better than the previous model, so all three of these variables were included in all later models. The inclusion of number of ideas before impasse (nIdeas) did not improve model fit, so was not included. We then tested the inclusion of distance, which did not independently improve model fit, direction which improved model fit and distance $\times$ direction which improved model fit. Finally, we tested for any interaction effects with distance $\times$ direction, and found a nearly significant interaction with Lit, but none with *Gf* or creativity.

**Fig. 4:**
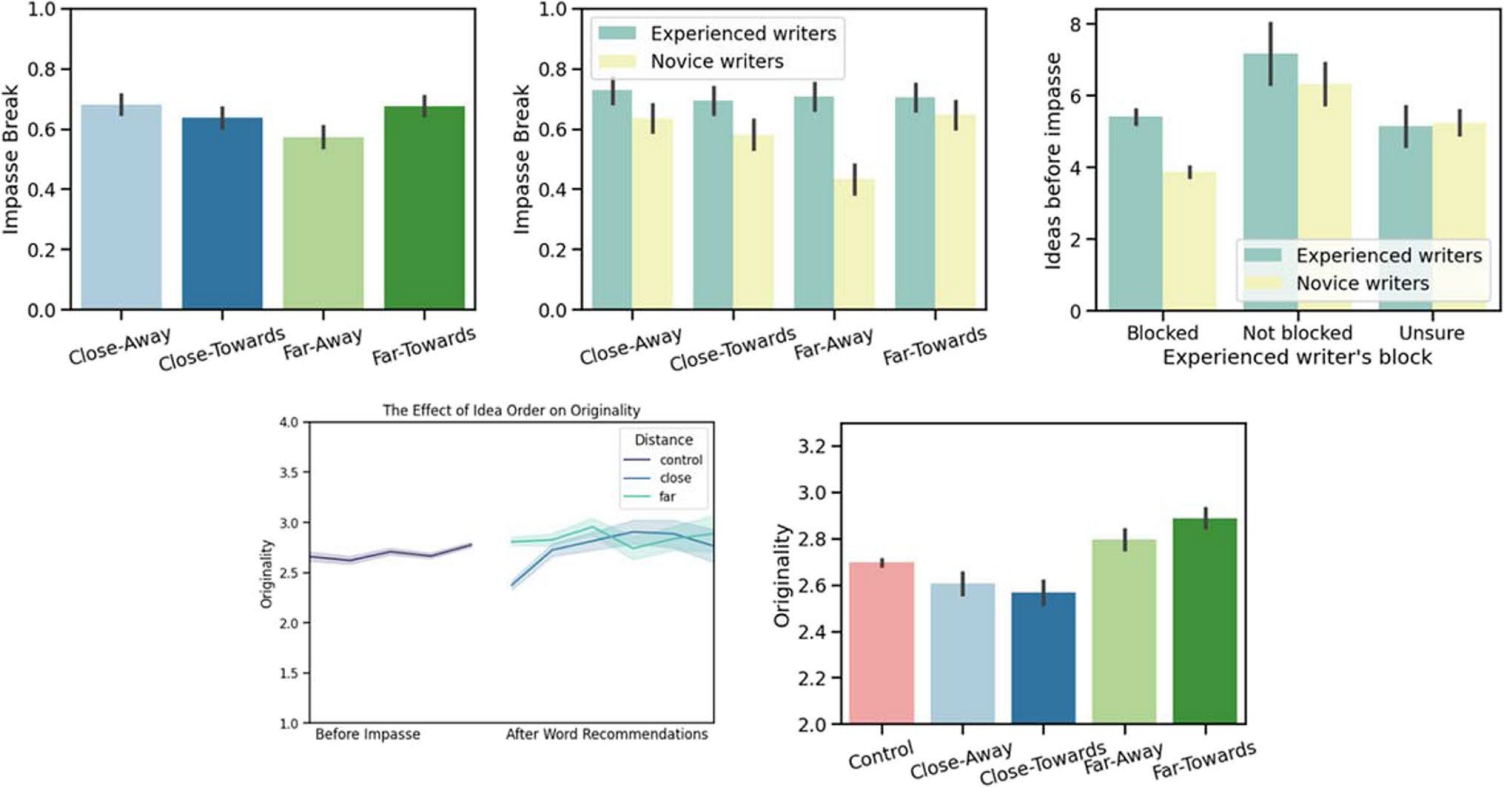
ACS impact on impasse break in Study 2. *Upper row*: impasse break. *Left:* impasse break according to word recommendation condition. *Upper middle*: Impasse break according to word recommendation condition, but further breaks this down according to writing experience. *Upper right*: How the novice and experienced writing groups differ in their subjective perception of writer’s block (according to how many pre-impasse responses it takes for them to attribute writer’s block to a given trial). *Lower left*: The effects of idea order and distance on response originality. *Lower right*: Originality according to word recommendation condition

The final model had significant effects of creativity (Est = 0.044, SE = 0.021, *p* = .036), and distance $\times$ direction: The far-away condition resulted in less impasse break than the other three word-recommendation types. Compared to far-away recommendations, far-towards had Est = 0.112, SE= 0.041, *p* = .006 more impasse break, close-towards had Est = 0.115, SE = 0.042, *p* = 0.005 more impasse break, and the relationship to close-away recommendations was nearly significant with Est = 0.078, SE = 0.041, *p* = .061 more impasse break (Fig. 4).

Because the interaction of distance $\times$ direction with writing fit our a priori hypothesis we also examined this model, and in addition to the effects described above, we also found that distance $\times$ direction effect was moderated by literature experience and was different in the far-away condition from the other word recommendation conditions. This was significant for the far-towards condition Est = – 0.202, SE = 0.083, *p* = .015, and nearly significant for close-away and close-towards. This effect can be seen in Fig. 4, where we see that the writing recommendation condition did not affect impasse break in the experienced writing group but had a large effect on the novice writer group who had an especially difficult time with impasse break in the far-away condition compared to the other conditions.

The final model in Study 2 did not reproduce the main effect found in Study 1 of *Gf.* However, *Gf* was nonetheless found to significantly improve model fit in the model analysis process, which accords with the finding in Study 1.

Next, we examine the impact of both fixed and random effects on the originality of responses, as per Study 1, with writing experience included as a fixed effect. After removing trials which did not have any responses after impasse, those with technical issues, and responses that clearly did not fit instructions we include 3345 pre-impasse and 1210 post word-recommendation responses in this analysis (Table 5).

**Table 5:** Chi-square difference tests for *originality*

Model parameters	Log-likelihood	AIC	${\chi }^{2}$	*df*	*p* value
Baseline	– 4964.2	9934.3	NA	NA	
Trial order	– 4963.2	9934.3	2.01	1	.156
Trial object (T)	– 4960.9	9929.8	6.52	1	.010
T + *Gf*	– 4960.3	9930.6	1.25	1	.262
T + C	– 4958.0	9926.0	5.83	1	.015
T + C + Lit	– 4957.7	9927.4	0.59	1	.442
T + C + Idea Order (IO)	– 4936.7	9885.4	42.56	1	< .001
T + C + IO + Distance	– 4920.3	9856.7	32.75	2	< .001
T + C + IO + Direction	– 4928.7	9873.4	16.05	2	< .001
T + C + IO + Distance * Direction	– 4913.8	9847.6	45.77	4	< .001
T + IO + Distance * Direction * C	– 4913.0	9854.1	1.56	4	.814
T + C + IO + Distance * Direction * Lit	– 4911.6	9853.2	4.45	5	.485
Baseline model: *Originality ~ 1 + (1| subject ID)*				${R}_{m}^{2}$	${R}_{c}^{2}$
Final model: Originality ~ (1|T) + C + IO + Distance * Direction + (1| subject ID)				.042	.267

For the final model, we report the marginal and conditional *R*^2^ values (Nakagawa & Schielzeth, 2013), which show the variance explained by the fixed effects (marginal), and entire model including both random and fixed effects (conditional) respectively

The baseline model included participants ID as a random effect. Similarly to Study 1, for originality, trial order was not included in the baseline because it had standard deviation and variances of 0 when included as a random effect, so it was tested later as a fixed effect. No demographic variables (age, sex, education) significantly improved model fit over the baseline. We then tested the effect of trial order as a fixed effect and did not find a significant improvement over baseline. Trial object (T) was then included as a random effect and did significantly improve model fit, so later models are compared to the trial object model. We then tested fixed effects. Adding *Gf* did not improve model fit. Creativity did improve model fit compared to the previous best model (T) so was kept for all future models and model comparisons. Literature experience did not improve model fit. Idea order did improve model fit compared to previous best model. Both distance and direction improved model fit compared to the previous best model, as did the interaction of distance and direction. The interaction of distance and direction with creativity and literature experience did not improve model fit.

In the final model the random effects were participants ID, Var = 0.099, SD = 0.316, and trial object, Var = 0.001, SD = 0.031. As in Study 1, the final model has significant effects for Idea order (IO), Est = 0.022, SE = 0.003, *p* < .001, and for distance $\times$ direction. In addition, in Study 2 the final model had a significant effect for creativity, Est = 0.051, SE = 0.019, *p* = .007. For distance $\times$ direction, Study 2 reproduced the effect found in Study 1 where the responses after close-towards recommendations where significantly less original than pre-impasse control Est = – 0.201, SE = 0.045, *p* < .001, and close-towards responses where significantly less original than far-towards, Est = – 0.312, SE = 0.059, *p* < .001. In addition, in Study 2 we found that the close-away responses were also less original than control, Est = – 0.188, SE = 0.045, *p* < .001, far-away responses were less original than control Est = – 0.098, SE = 0.046, *p* = .034, and far-towards responses were more original than control Est = 0.110, SE = 0.046, *p* = .017. We also found that far-towards responses where the most original of all the word recommendations: In addition to being more original than pre-impasse control, and close-towards responses (as shown above) the far-towards responses were also more original than close-away, Est = 0.299, SE = 0.059, *p* < .001 and far-away responses, Est = 0.209, SE = 0.057, *p* < .001.

Overall, these results replicate the main effects found in Study 1. In both cases idea order (IO) had a positive effect on originality, and close-towards responses were less original than pre-impasse control responses, and less original than far-towards responses.

Next, we examined how writing expertise affects self-perception of impasse break and response originality. In Study 2, participants were asked a series of questions following the five ACS trials evaluating the perceived utility of the word recommendations. These questions include whether the participant experienced writer’s block during the tasks; self-rated originality before and after the word recommendations; and whether the word recommendations helped spark new ideas. We measured the correlation between reported experience of writer’s block, participant’s subjective reported originality of responses, and fluency. These correlations were analyzed for the novice and expert groups using Spearman correlations, and the Benjamini and Yekutieli multi-test analysis to reduce false discovery rate was applied (Benjamini & Yekutieli, 2001). We found that novice writers associate writer’s block with fluency (number of ideas before impasse), Novice: *r* = – .24, *p* < .001, Experienced: *r* = – .07, *p* = .751, while experienced writers associate writer’s block with a perceived increase in originality after the word recommendations, Novice: *r* = .150, *p* < .068, Experienced: *r* = .232, *p* < .001 (Fig. 4).

**Fig. 5:**
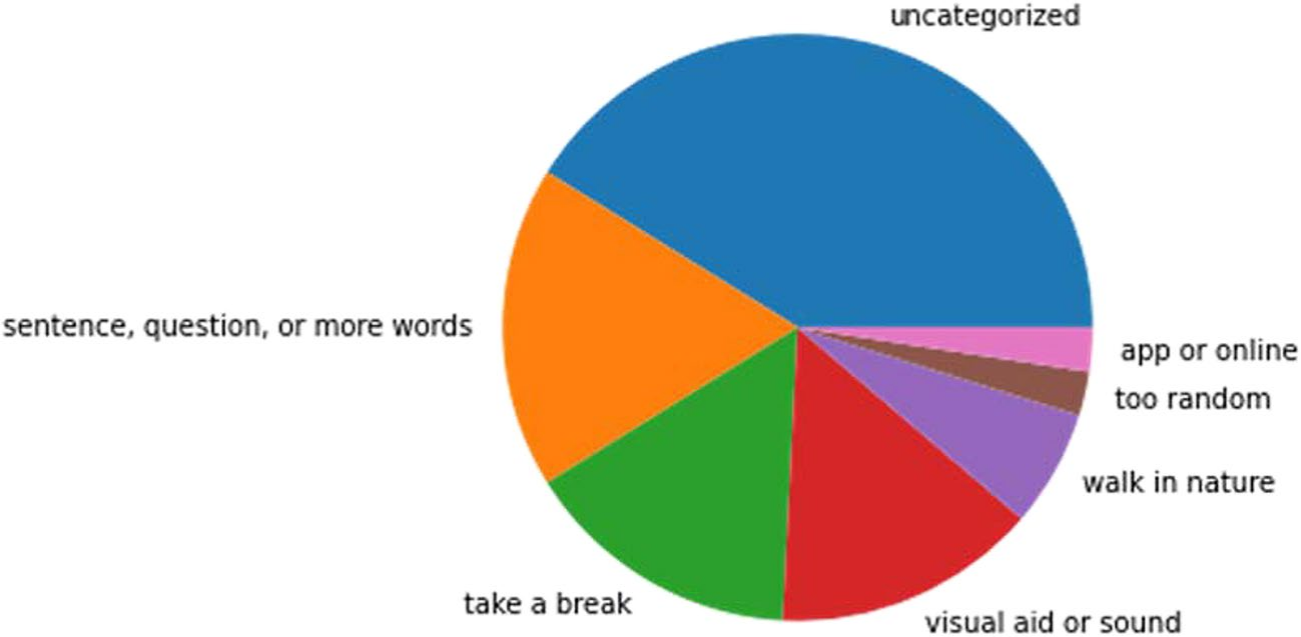
Themes in participant feedback to the question: “Do you have any suggestions or feedback for us about what you think would help solve writer's block and aid in creative writing tasks?”

Finally, we qualitatively analyzed participants’ feedback about the ACS (questions listed in Appendix A). We found that 76% of participants reported experiencing writer’s block during the tasks. 37% of participants reported the word recommendations to be helpful, and 23% reported them as ‘neutral.’ When asked if and how the word recommendations helped some participants reported that the word recommendations were either “too obviously related to the subject” or to [their] “previous ideas” to be helpful, or “utterly bizarre” and too disconnected from the subject to prompt creative ideas, suggesting that participants are sensitive to effects of semantic distance of the word recommendations (Fig. 5). However, others reported using the word recommendations to form new and creative associations. For example, one participant wrote “Yes, it was surprising how I had gotten myself stuck in a corner, but when the new word was given, I suddenly saw some completely new avenues to investigate, the shoe being the first and then, love. I immediately thought of a horseshoe and that changed everything.”

We then analyzed participants’ suggestions for improving the ACS. First, we evaluated participants’ answers and used a grounded theory method to identify common themes. We then searched the answers for the keywords representing each of the themes. Many participants requested to see more word recommendations for each task, longer phrase suggestions or questions (Fig. 5). Some also suggested more experiential writer’s block help such as music or having the participant take a break or walk in nature.

### Discussion

Study 2 aimed at replicating and extending the results found in Study 1, demonstrating the success of the ACS in breaking participants’ mental fixation. Study 2 goes a step further by examining how literature experience relates to the success of the ACS. In Study 2, participants had at least one response to the word recommendations in 64% of trials, and 95% had a response after the word recommendation at least once. In the experienced writing group 70% (95% CI 66.5–77.9 %) of trials had responses after impasse, while in the novice group it was 58% (95% CI 52.8–61.9%), reproducing the effect found in Study 1. The rate of impasse break is almost twice as high as in Study 1, which might be attributable to the preliminary filtering resulting in a group more motivated to complete creative writing tasks. Taylor and Barbot (2024), found a positive correlation between creativity and effort in a story writing task and in a divergent thinking task (AUT), supporting the notion that the group of people who indicated interest in an experiment related to creative writing would put more effort into the ACS task, as opposed to the unfiltered population sampled in Study 1.

This finding was confirmed in the statistical analysis of the impasse break model, which found that literature experience significantly improved model fit. We found a nearly significant effect of the far-away condition negatively impacted the likelihood of impasse break for novice writers, but not for experienced writers (Fig. 4; bottom left). This effect of literature experience thus moderated the negative effect of far-away word recommendations on impasse break found in the final model. While this effect was not found in the statistical analysis of Study 1, a post hoc analysis showed this same pattern as in Study 1. However, we did not find significant effects of literature experience on originality (same as Study 1).

Overall, the effects of distance and direction replicated and expanded on the results found in Study 1. In both studies, close-towards responses were significantly less original than control (pre-impasse) responses and were less original than far-towards responses. In Study 2, we further found that far-towards responses were more original than the pre-impasse control responses and the three other word recommendation conditions. Study 2 also reproduced the effects of idea order on response originality found in Study 1.

## General discussion

In the current study, we introduce the Associative Creativity Sparker (ACS), which we use to investigate the role of semantic distance and semantic relatedness to previously explored ideas (direction) of text recommendations on impasse break and originality. The ACS draws on cognitive network science (Siew et al., 2019) to enable the investigation of effects of word recommendations at different locations in semantic memory on idea generation and impasse break. We also examine the roles of *Gf*, creativity, and literature experience on the utility of word recommendations. This allowed us to ask how different types of text recommendations might provide greater utility to people with differing cognitive traits, with implications for the design of human machine co-creativity tools.

Our findings indicate that the ACS can be a useful method for breaking participants out of mental fixation during creative ideation and divergent thinking. This may facilitate a greater understanding of creative fixations and blocks (Smith, 1995), including writer’s block by using the ACS recommendations during impasse states to examine what factors break participants out of creative block. Furthermore, the ACS allows us to study the co-creative process where a person completes creative tasks with a machine in the loop (Rafner et al., 2023), and tie our theoretical understanding of co-creativity to new insights into the creative process stemming from semantic network analysis.

We conducted two studies with the ACS: in Study 1 we find that the ACS is effective at breaking participants out of creative fixations, that it increases the originality of responses compared to pre-impasse responses, and that the experimental parameters of the word recommendations significantly affect the originality of responses. In Study 2, we replicate these results and also show differences in impasse break between novice and expert writer’s using the tool. These results demonstrate the ability of the ACS in manipulating the process of creative association (Beaty & Kenett, 2023), or spreading activation of concepts (Collins & Loftus, 1975), and to our knowledge is the first tool explicitly aimed at facilitating the study of the role of semantic memory in creative ideation and creative block, by directly activating specific areas in semantic memory.

Overall, in this work we find that the interaction of distance $\times$ direction of word recommendations affects the likelihood of impasse break. Far-away recommendations are the least likely to result in impasse break (Study 2). Semantically farther recommendations are less likely to result in impasse break than semantically close recommendations. However, this negative effect of *distance* is attenuated by the *direction* of the word recommendations towards or away from the participants previous ideas, and is found to interact with writing expertise: in Study 2 we find that novice writers struggle to draw on far-away recommendations, but when far-towards recommendations are shown, which are equally far but more closely related to ideas the participant already considered, the likelihood of impasse break is not negatively affected. This interaction effect with literature experience was nearly significant in Study 2, and evident from the data but not significant in the model of Study 1, likely because of lack of power. Notably, fluency in the pre-impasse section of the ACS – equivalent to the fluency measure in a standard AUT (Acar & Runco, 2019) – did not predict the likelihood of impasse break for Study 1 or 2.

This effect demonstrating that far-away word recommendations are more difficult to use suggests that participants may be exploring the semantic area around the word recommendation through a spreading activation process (Collins & Loftus, 1975; Siew, 2019), and it is harder to spark usable associations to distant and unrelated concepts. This could be tied to theories of foraging in semantic memory (Hills et al., 2012), which suggest that humans use optimal and dynamic search strategies to find semantically related concepts, switching semantic ‘patches’ once the rate of finding new concepts in the current ‘patch’ becomes too slow (Todd & Hills, 2020). Related to our findings, Ovando-Tellez, Benedek et al. (2022a), Ovando-Tellez Kenett et al. (2022b) have shown how switching between clusters is related to a richer semantic memory structure. In addition, Beaty et al. (2023) demonstrate how the semantic neighborhoods of AUT objects impact the quantity and fluency of participants’ responses, and how such effects impact serial order of idea originality and interacts with *Gf*. Finally, Marko et al. (Marko & Riečanský, 2021; Michalko et al., 2023) have shown how semantic neighborhoods (or communities) impact retrieval of words in a semantic fluency task.

Taken together, the effects on impasse break and originality seem to indicate that the responses after the word recommendations were not simply a continuation of the participants’ idea generation process before reaching a block. Rather, the word recommendations encourage participants to explore previously unconsidered areas in their semantic memory. The fact that distance and direction of the word recommendations affect the likelihood of impasse break, shows that participants to at least some extent attempt to form associations to the concept recommended to them, and are sensitive to its content.

The effects of distance and direction on originality contrast with the effects found on impasse break. In both studies, we found that close-towards responses are the least original, and Study 2 showed that far-towards responses are the most original, with far-away and close-away responses somewhere in the middle. This further suggests that the ACS sparks a semantic memory search, as participants may have the easiest time accessing concepts in areas that are semantically close to the object, and connected (towards) the ideas they already had, but many of these concepts may have already been visited and the remaining ones inspire the least original ideas of all. The far-away responses, in contrast, may have been too distant in semantic memory for participants to adequately explore, causing these to result in the least amount of impasse break, while the far-towards responses which are sufficiently semantically far from the trial object, but close enough to the participants’ previous ideas to be explorable led to more original ideas.

It may be that especially original associations are sparked when participants use the word recommendation as the starting point for a search in semantic memory, rather than fixating on the specific concept suggested, also further highlighting the significance of idea remoteness in creative thinking (Benedek et al., 2023). This may correspond to the adversarial use of large language models described in Calderwood et al. (2020), where novelists use the recommendations as ideas of what not to write.

Finally, both studies reproduce the serial order effect (Bai et al., 2021; Beaty & Silvia, 2012). In the pre-impasse section ideas become more original over time, and even after seeing the word recommendations the ideas start from roughly the level of originality at impasse and continue to increase. However, as seen in Figs. 3 and 4, in the far condition, the originality of ideas reduces over time, while in the close condition it goes up, suggesting that participants may be able to continually explore outwards from close recommendations, while the challenge of far recommendations causes them to fixate again. This shows a possible effect interaction between the structure of activated concepts in semantic memory and the serial order effect, which was not found in previous work examining the interaction between serial order and target object set size (Beaty et al., 2023).

In contrast to the negative effects on impasse break of far-away recommendations, we find that these recommendations have a positive effect on originality of responses. This may indicate a trade-off between fluency and originality of responses after the word recommendation in the ACS, echoing the trade-off effect between AUT responses to rich semantic cues (with a large degree of edges in the semantic memory network) and sparse semantic cues found in Beaty et al. (2023). In our case, while we do not take into account the effects of word recommendation or target object degree (neighborhood set size), it could be that the distance and direction parameters similarly affect the number of possible associations a participant is able to make between the target object and word recommendation.

Because the structure of semantic memory networks has been found to vary in connectivity and flexibility according to creativity and *Gf* (Kenett, Beaty et al., 2016a), we hypothesized that these variables would affect impasse break and originality. We found mild support for this hypothesis. *Gf* significantly affected impasse break in both studies, creativity significantly affected both impasse break and originality in Study 2, and literature experience significantly affected impasse break in Study 2. Moreover, we hypothesized that there would be interaction effects of the distance and direction parameters with *Gf*, participant creativity and literature experience. However, we did not find this effect. We did find a nearly significant effect of writing experience and distance x direction in Study 2, partially supporting this hypothesis.

Generally, our work deals with a very common phenomena, of creative block. It is a frequent part of people’s creative process, and is a source of negative emotion, distress and even loss of income (Ahmed & Güss, 2022). Previous work attempting to mitigate creative block have examined various methods such as group brainstorming, crowdsource suggestions, sleeping and incubation periods, subliminal messaging, and hypnotically inducing dreams (Chan et al., 2017; Davé, 1979; Gonçalves et al., 2017; Nijstad & Stroebe, 2006; Siangliulue et al., 2015; Sio et al., 2013; Sio & Rudowicz, 2007). Yet, these efforts target creative thinking in general, less focusing on specific stages of the creative process (Benedek et al., 2023). The rapid development of computational methods over the past few years is leading to an increase in the development of text-based creativity support systems (Calderwood et al., 2020; Chan et al., 2017; Huang et al., 2020; Kang et al., 2022; Lee et al., 2022; Rhys Cox et al., 2021; Siangliulue et al., 2015). Our current work on the ACS builds on and extends this work, by integrating cognitive theory from creativity research with computational methods from cognitive network science, text analysis, and recommendation systems. A significant characteristic of the ACS is its modular structure, whereas each of its components (e.g., semantic memory network model, text analysis method, and word recommendation method) can be easily swapped with corresponding components. Thus, the ACS offers a general basis for many possible future expansions and adaptations.

## Limitations

Several limitations exist in our study. First, in the ACS itself, basing word recommendations on an aggregated representation of semantic memory means that the location of word recommendations is only roughly approximated. Future work is needed to examine how more individualized representations of semantic memory might improve results and reduce noise (Benedek et al., 2017; He et al., 2020; Ovando-Tellez, Kenett et al., 2022b). However, the question of how to generate individual semantic networks on a large enough scale to be useful for this type of study is still open (e.g., Wulff et al., 2022).

Second, while we used a simplified measure for the distance and direction parameters, it is imperative to recognize the inherent limitation concerning the uniform importance assigned to all words. Although our approach acknowledges this limitation through stoplisting, a more nuanced strategy such as employing term weighting to adjust network edge weights could have potentially enhanced the accuracy of direction, distance, and adherence scores by mitigating noise. This adjustment is suggested as a consideration for future work using the ACS system.

Another limitation relates to our approach in measuring literature expertise in Study 2. We note that due to the sample available on Prolific, the experienced group in our study is likely not composed of true writing experts, but rather of slightly more experienced writers than the average person. In addition, because participants completed the entire ICAA questionnaire again in the main study, we were able to compare responses to the literature section in the preliminary and main survey and found high variability in responses between the two sets of responses. This was especially notable in the expert group: some participants may have assumed that if they report greater writing experience, they would be more likely to be chosen to complete the follow-up study. However, even in the novice group a high variability can be seen between the two runs. For literary achievement there is a correlation between the two runs of .26 in the novice group and .32 in the experienced group; for literary activity the correlation is .53 for the novice group and .70 for the experienced group. This may point to some difficulty in accurately answering the ICAA questionnaire. Because of the low number of participants who had exactly the same answer in both questionnaires, we include all participants in our final analysis. The lack of truly expert writers in Study 2 may also affect results. Future studies more reliably estimating novice and expert writing groups that are more different from each other in experience level might demonstrate different behaviors than the groups we used.

In future directions, the ACS may be used to examine other aspects of co-creativity and the creative ideation process apart from writer’s block, as was examined in this work. For example, a word recommendation might be shown several times in the same trial, or once after every response. This can allow examining whether people may be slowly and iteratively guided by the text recommendations towards especially creative and distant areas in semantic memory. Many participants in our study requested that the tool show more than word recommendation per trial, or show phrases, questions, or images instead of single words – these options were identified by participants as potentially useful for real-life creative writing endeavors. Other modifications to the tool or study design may be made to bridge from this current setup to real life co-creative and creativity support tool scenarios. For example, it is possible to adjust the tool so that participants write part of a short story, or give ideas for slogans, metaphors or jokes and request assistance when experiencing writer’s block. The word recommendations themselves could also be modified to be phrases by prompting a large language model to generate phrases based on the concept in the location in semantic memory generated by the original word recommendation algorithm. Finally, we add that this tool is configured to be usable for neuroscience research using EEG or fMRI, as it runs on any computer and collects timing data for every action of the participant. In addition, it can be applied in different populations, both healthy and clinical, that exhibit increased rigidity of thought – such as shown in older adults or people with high-functioning autism (Cosgrove et al., 2021; Faust & Kenett, 2014; Kenett, Gold et al., 2016b).

## Conclusions

In conclusion, our research introduces the Associative Concept Sparker, which allows us to study creative fixation, by giving participants personalized word recommendations at various locations in semantic memory at the time they experience writer’s block in a divergent thinking task. Our research demonstrates that the ACS is often effective at breaking participants out of writer’s block. Responses after seeing the word recommendations are often more original than responses before impasse, but this also has a tradeoff with impasse break: word recommendations that are too distant from the target object and previously considered responses may inspire the most creative responses but are least likely to inspire new ideas. Yet, we find that this effect may differ between novice and expert writers.

As such, our research demonstrates how current understanding of the role of semantic memory in the creative process facilitates generating remote associations that can be applied to “spark” new ideas and break mental impasse (Beaty & Kenett, 2023; Benedek et al., 2023; Kenett, 2024). Furthermore, our work highlights an exciting new era of research, one that focuses on co-creativity tool development, and how personalizing such tools can facilitate creative impasse breaking.

## Data Availability

Data and materials are available at https://github.com/TaliaWise/ACS/tree/main

## Appendix

### Writer’s block questionnaire

1. How much did the word suggestions help you come up with new ideas?

(Likert scale: 1 Not at all, … 5: Very much)

2. How creative would you rate the ideas you had in each task before you ran out of ideas and saw the word suggestions?

(Likert scale: 1: Not at all creative …. 5: Very creative)

3. How creative would you rate the ideas you had after you saw the word suggestions?

(Likert scale: 1: Not at all creative …. 5: Very creative)

4. Did you like getting the word suggestions?

(Likert scale: 1 Not at all, … 5: Very much)

5. Did you experience writer's block while completing these tasks?

(Yes, No, Unsure)

The next few questions refer to your writing experience in your daily life. If you do not ever write please select the IRRELEVANT option for these three questions.

6. How often do you experience writer's block when you write?

(Irrelevant - I do not write, or Likert scale 1: Never, … 7: Always)

7. What causes writer's block for you? Select all that apply:

(fear of evaluation, perfectionism, procrastination, writing anxiety, decreased intrinsic, motivation, overplanning, inconsistent writing habit, interruptions, other (please type),

IRRELEVANT: I do not write or do not experience writer's block)

8. What do you do to solve writer's block when you experience it?

(take a break, work on a different project, force myself to keep writing, other (please type), discuss ideas with others, IRRELEVANT: I do not write or do not experience writer's block)

9. Did you feel the word suggestions in these tasks helped you come up with new ideas? If so, how?

(open text answer)

10. Do you have any suggestions or feedback for us about what you think would help solve writer's block and aid in creative writing tasks?

(open text answer)
